# Characterisation of the bacterial and archaeal microbiota in fresh colostrum collected from a single, spring-calving dairy herd

**DOI:** 10.1371/journal.pone.0335718

**Published:** 2025-10-30

**Authors:** Sabine Scully, Bernadette Earley, Paul E. Smith, Matthew S. J. Finnie, Catherine McAloon, Frank Buckley, David A. Kenny, Sinéad M. Waters

**Affiliations:** 1 Animal and Bioscience Research Department, Animal and Grassland Research and Innovation Centre, Teagasc Grange, Dunsany, Co. Meath, Ireland; 2 School of Veterinary Medicine, University College Dublin, Belfield, Dublin, Ireland; 3 Animal and Bioscience Research Department, Animal and Grassland Research and Innovation Centre, Teagasc Moorepark, Fermoy, Co. Cork, Ireland; 4 School of Biological, Earth and Environmental Sciences, University College Cork, Co. Cork, Ireland; IZ: Instituto de Zootecnia, BRAZIL

## Abstract

There is increasing evidence to support the existence of a naturally occurring colostral microbiome, which may influence the development of the gastrointestinal microbiota and immune function of the calf. The objective of this study was to characterize the prokaryotic community of colostrum collected fresh (within 2h parturition) from primi- and multiparous Holstein-Friesian (n = 17) and Jersey (n = 10) cows. Extracted microbial DNA underwent qPCR and Illumina amplicon sequencing of the V4 region of the 16S rRNA gene. High throughput amplicon sequencing data was analysed using a variety of *R* packages. Taxonomy was assigned using the SILVA database (v. 138.1). No effect of breed or parity was observed on alpha (α; Shannon) diversity and community composition. The mean Shannon score was 3.33 (SE 0.14), indicating a diverse community within sample. A total of 681 genus-level amplicon sequence variant (ASV) groups were identified prior to filtering for relative abundance (RA) of >0.05%. Nineteen bacterial genera were identified as core. The predominant bacterial phyla observed were *Bacillota, Pseudomonadota,* and *Actinomycetota*. Community membership consisted of common gut commensals, with many members exhibiting diverse metabolic functions. Within the archaeal community, *Methanobrevibacter* had the highest RA*,* accounting for 85.99%. No observed differences between breeds suggests that farm origin may be more influential than breed on microbiota composition. The presence of archaea and strict anaerobes highlights the need to investigate the existence of an entero-mammary pathway in cattle. This is the first study jointly characterising bacteria and archaea in colostrum from different breeds from the same dairy herd under pasture-based conditions. The diverse bacterial community observed warrants further investigation into its role in calf health in early life. Specific microbes, like *Lachnospiraceae*, should be investigated for their potential in the development of probiotics and preventative practices for better calf health.

## 1. Introduction

Bovine colostrum contains many bioactive components, such as immunoglobulins (Igs), energy-rich nutrients (fats, proteins, etc.), hormones and growth factors, microRNAs, antimicrobial peptides, and microorganisms that are essential for the immune and physiological development of the neonatal calf [1]. The syndesmochorial placenta of cattle does not allow for the intrauterine exchange of Ig antibodies and other important macromolecules during gestation [2]. Consequently, calves are born immunologically naïve and depend on colostrum consumption post-calving to obtain passive immunity through the transfer of Igs and other bioactive compounds [3]. Recent research has begun to look beyond immunoglobulins, examining other colostral bioactive components, including microbes, to better understand colostrum as a matrix and its role in calf health and development. There is increasing evidence of a colostral microbiome, which is not the result of environmental contamination [4]. Studies done in humans, pigs, and mice support the existence of an entero-mammary pathway, where gastrointestinal (GIT) microbes are mobilized and transferred from the gut to the mammary glands during colostrogenesis and later milk production [4–5]. This link has not yet been established in ruminants, however, the presence of strict anaerobes in bovine colostrum and milk indicates deeper complexities than previously suggested [6–7]. These pioneering microbes are essential to GIT microbiome and immune development of the neonatal calf [8–9]. The GIT microbiome plays a crucial role in immune development, as the commensal GIT microbiota and their metabolites are vital for the development of the mucosal barrier [8,10]. The origins of the pioneering microbes that colonize the calf GIT remain a topic of debate, yet there is consensus that both the dam (maternal sources) and the environment play a role in seeding and colonising the calf’s microbiome [9,11].

Exploration of the colostral microbiota in association with microbial seeding [12–15], mastitis [6], dry cow management [16] and calf immunity/health status [17–18] has provided deeper insights into the complexities of bovine milk and colostrum. For example, an increased presence of *Staphylococcus, Mycoplasma* and *Streptococcus* in colostrum has been associated with clinical mastitis in lactating cows [6]. Compositional differences between primi- and multiparous colostrum donors has also been reported [6], however, this not been replicated in other colostrum microbiome studies [16,18]. The colostral microbiota of beef and dairy cattle differ, and an effect of season has been reported [18]. A link between specific colostral bacteria in dairy cow colostrum and the passive immune status of dairy calves was also observed. Although Van Hese et al. [18] reported compositional differences between the colostral microbiota of Holstein-Friesian and Belgian Blue breeds, these differences were not attributed to breed. These animals were sourced from a dairy and beef herd from the same research farm but reared under different types of management systems. In contrast, other studies examined the colostral microbiota of Holstein cows, from intensive commercial dairy production systems, where animals were housed indoors year-round [6,16,19].

While it is well established that farm origin and management practices play a significant role in the development of bovine microbial communities [20–21], to the authors’ knowledge, no published research has examined the colostral microbiota of different dairy breeds, or the colostral microbiota of dairy cows raised and managed within a pasture-based production system. Additionally, no study has jointly characterised the bacterial and archaeal components of fresh colostrum of different breeds reared under the same production system. In order to better understand the complexities of the colostral microbiota in dairy cows, the objective of this study was to characterize the Ig quality, archaeal and bacterial components of fresh colostrum from spring-calving primi- and multiparous Holstein-Friesian and Jersey cows raised and managed within the same pasture-based dairy production herd.

## 2. Materials and methods

### 2.1 Ethics statement

The experiment was undertaken at the Dairygold Research Farm in Kilworth, Co. Cork, Ireland (Teagasc, Animal and Grassland Research and Innovation Centre, Moorepark, Fermoy, Co. Cork, Ireland; 52°09’N; 8°16’W). All animal procedures performed in this study were undertaken by trained research personnel, were approved by the Teagasc Animal Ethics Committee, and are consistent with the experimental license (AE19132/P148) issued by the Irish Health Products Regulatory Authority under European Union legislation (Directive 2010/63/EU) for the protection of animals used for scientific purposes.

### 2.2 Study design and animal model

#### 2.2.1 Colostrum donors and management.

Colostrum was sourced from a primi- (n = 10) and multiparous (n = 17) (1–10 lactations, mean lactation 2.93 (SE 0.46)) herd consisting of Holstein-Friesian (HO; n = 17) and Jersey (JE; n = 10) cattle (Table 1) all of which were raised and managed within a single, spring-calving system.

**Table 1:** Sample population frequencies by breed and parity.

	Primiparous	Multiparous	Total
Holstein-Friesian	5	12	17
Jersey	5	5	10
Total	10	17	27

**Fig 1:**
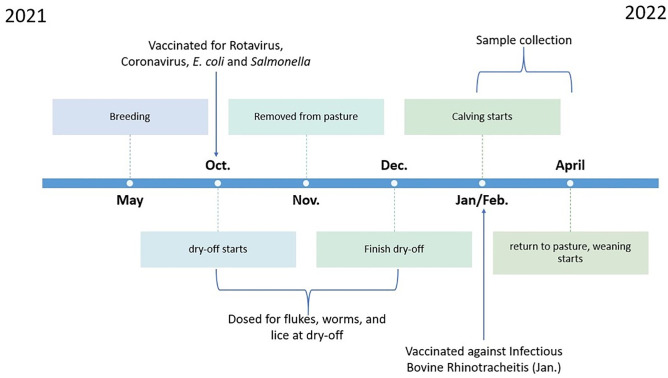
Dam management and sampling timeline.

Cows and heifers grazed perennial rye grass/white clover pastures until November 2021, after which they were accommodated indoors until approximately April 2022 (Fig 1). Pregnant heifers and cows were penned in the housing unit by reproductive status (heifers separate from cows), in a free-stall system fitted with rubber mats, slatted floors in the aisles and automated waste removal. While indoors, all cows and heifers were offered home-grown grass silage (perennial rye grass/white clover) *ad libitum* without any supplementary concentrate feed. Pregnant cows and heifers were vaccinated against Rotavirus, Bovine Coronavirus, and *Escherichia coli* (Bovilis® Rotavec®Corona, MSD Animal Health, Ireland); Infectious Bovine Rhinotracheitis (IBR; Rispoval® IBR-Marker inactivated, Zoetis Belgium S.A., Dublin, Ireland) and *Salmonella* (Bovilis® Bovivac S, Intervet Ireland Limited, Dublin, Ireland). At dry-off, cows and heifers were treated for internal and external parasites (Cydectin: 0.5% w/v pour-on for cattle, Zoetis Belgium S.A., Dublin, Ireland). Fourteen of the 17 cows had somatic cell counts greater than 100,000 and thus received an intramammary antibiotic (Ceravin Dry Cow 250 mg intramammary suspension, MSD Animal Health, Ireland) prior to teat sealing. All cows had teat sealant (Boviseal, Zoetis Belgium S.A., Dublin, Ireland) applied at dry-off. Heifers entering first calving and first lactation received no teat sealant nor udder treatment prior to calving. Based on the expected calving date and clinical signs, pregnant cows and heifers were moved from the main herd and housed in a separate calving unit approximately one week prior to parturition. Cows and heifers in the calving unit were accommodated on deep straw-bedded concrete floors and had *ad libitum* access to water and home-grown grass silage (perennial rye grass/white clover), with no supplementary concentrates provided.

#### 2.2.2 Colostrum sample and data collection.

Colostrum was milked from each mammary gland quarter of each cow within two hours of parturition, by research personnel, using a portable milking unit (MK100 034904 2017). The portable milker was washed with anti-microbial soap (Hygienic C032, bactericidal hand soap, Selden Research LTD., Buxton, Derbyshire, England) before and after each milking, then rinsed thoroughly with boiling water, in line with standard practice on commercial farms. Boiling water (100°C) was run through the unit after washing and prior to the milking of the next cow. The teat-cluster was sprayed with 70% ethanol/molecular-grade water solution and wiped clean after each wash and again prior to next use. For the colostrum donor, each teat was wiped clean, individual quadrants of the mammary were then stripped of its initial contents, sprayed with 70% ethanol/molecular-grade water solution, and wiped clean prior to application of the teat-cluster. All equipment used for collection, storage, transfer of colostrum and calf feeding were cleaned with anti-microbial soap after each use. All colostrum collected was considered “fresh”, meaning that it was collected directly from the dam and fed to the calf immediately after collection, without undergoing any storage or re-heating. At time of collection, colostrum quality was assessed using a digital BRIX refractometer (Hanna Digital Refractometer for Sugar, HI9681). BRIX refractometry is an accepted proxy used on farm and in veterinary science to assess colostrum quality, as it is an indirect measure of IgG. Using nitrile gloves that were sterilized with 70% ethanol/molecular-grade water solution, 30 millilitres (mL) of colostrum was aliquoted into three sterile 10 mL tubes. The samples were snap-frozen immediately by immersion in liquid nitrogen, kept on dry ice during sampling and transport to the laboratory, and stored at −80°C until further analysis.

### 2.3 Sample processing

#### 2.3.1 Colostrum pre-processing.

Colostrum pre-processing methodology was based on protocols described by Blans et al. [22] and Siebert et al. [23]. Colostrum sample processing order was determined on a random basis. Before centrifugation, the 27 × 10 mL colostrum aliquots were thawed by transferring from −80°C to −20°C for 24h, then to +4°C for 24h. Once thawed, 600 µL of 0.5M Tris EDTA pH8 buffer for molecular biology (Thermo Fisher Scientific, Belgium) was added to each 10 mL colostrum aliquot to remove caseins. The sample was vortexed to ensure complete mixing and then centrifuged at 4,500 × g for 20 minutes at +4°C. After centrifugation, the upper fat layer was removed and discarded; 6 mL of supernatant (the fat-free layer) was pipetted into three sterile 2 mL microfuge tubes. The colostrum supernatant was then stored at −80°C until single radial immunodiffusion (sRID) was performed. The colostrum pellet was transferred using a pipette tip to a 1.5 mL microfuge tube (Eppendorf LoBind) and stored at −80°C until nucleic acid extraction was performed.

#### 2.3.2 Single radial immunodiffusion.

Single radial immunodiffusion (sRID) analysis of the colostrum supernatants was conducted to quantify immunoglobulins (Ig) and determine colostrum quality [24]. In brief, quantification of IgA, IgM and total IgG were performed using commercially available sRID kits (radial immunodiffusion test for quantification of bovine IgA, IgM and total IgG in serum or plasma, (Triple J Farms, Bellingham, Washington, USA)). Each of the sRID plates was supplied with three standard controls (data available on Open Science Framework (OSF)) and 5 µL of each was applied to the respective plates. Colostrum supernatant samples were diluted with 0.9% NaCl (1:6 dilution) to fall within the range of the standard curve, and 5 µL of each diluted colostrum sample was applied to the sRID plates.

#### 2.3.3 Extraction, qPCR and amplification of the colostral microbial DNA.

DNA was extracted from the colostrum centrifugation pellet samples via repeated bead-beating and silica spin column purification using the Qiagen DNeasy® PowerSoil® Pro kit (Qiagen, Manchester, England, United Kingdom) as described previously [24–25]. Extractions were performed in batches of 12, which consisted of 11 colostrum centrifugation pellets, and one negative extraction control (i.e., an extraction performed without a sample). To monitor the DNA extraction efficiency, three DNA extractions from each 50-sample extraction kit were also performed to extract DNA from the ZymoBIOMICS™ Microbial Community Standard (Zymo Research Corp., Irvine, California, USA). Concentration and purity of extracted DNA was quantified on the Nanodrop 1000 Spectrophotometer (Thermo Fischer Scientific) and high molecular weight DNA integrity was assessed by electrophoresis on 0.8% agarose gels.

DNA samples extracted from colostrum were submitted to 16S quantitative polymerase chain reaction (qPCR) analysis in order to verify the presence of bacterial DNA prior to 16S rRNA gene amplicon sequencing. The analysis was performed using 27 colostrum samples, six negative controls (molecular biology grade water) and three *Staphylococcus* positive controls. For each qPCR reaction, 1 µL of DNA sample (10 ng/µL) was added to the bottom of a well in a 96-well plate, followed by the addition of 19 µL of qPCR master mix. The resulting mixture was gently pipetted up and down six times. Each 19 µL of master mix consisted of 10 µL of Fast SYBR (Life Technologies, UK), 7 µL molecular biology grade water (Sigma, UK), 1 µL of forward primer Ba27F 5’AGA GTT TGA TCC TGG CTC AG (5uM), 1 µL reverse primer Ba338R 5’TGC TGC CTC CCG TAG GAG T (5uM) [26]. The qPCR reaction was performed on an ABI7500 FAST qPCR machine (Applied Biosystems, UK) with 7500 Fast Software v2.3. Reaction conditions were 95°C for 20 seconds, then 40 cycles of 95°C for 3 seconds and 60°C for 30 seconds. After completion of the qPCR run, the cycle threshold was set to 0.2 and baseline was set to automatic then CQ values were calculated. These CQ values were then used to verify the presence of bacteria within the colostrum samples selected for 16S rRNA gene amplicon sequencing. One colostrum sample and two negative controls came back as undetermined. Fresh colostrum had a mean CQ value of 23.5 (SE 0.87), ranging from 17.83 to 26.46. Negative controls had a mean CQ value of 36.73 (SE 0.16) and the *Staphylococcus* positive controls had a mean CQ value of 12.30 (SE 0.15). The CQ values of fresh colostrum attained from qPCR verified that, while low in biomass, bacteria were present in fresh colostrum in adequate amounts for sequencing.

Aliquots of DNA were subjected to 16S rRNA gene amplicon library preparation and sequencing at a commercial laboratory (Macrogen, Seoul, South Korea). Samples underwent one round of PCR amplification, targeting the V4 hypervariable region of the 16S rRNA gene, using 515F/806R primers [27] designed with Nextera overhang adapters and Herculase II Fusion DNA polymerase (Agilent, Santa Clara, California, USA). Cycle conditions were as follows: 95°C for 3 min, 25 cycles at 95°C for 30 s, 55°C for 30 s, 72°C for 30 s, and then 72°C for 10 min. PCR amplicon purification was performed using standard AMpure paramagnetic bead protocol (Beckman Coulter, Indianapolis, Indiana, USA). Amplicons were pooled together in equal concentration and subjected to sequencing on the Illumina MiSeq using the 500-cycle version 2 MiSeq reagents kit (Illumina, San Diego, California, USA) on one flow cell.

### 2.4 Sequencing analysis

Amplicon sequencing data were processed in *R* (v. 4.2.0) using *DADA2* (v. 1.26.0) and submitted to the pipeline as described by Callahan et al. [28] following a similar methodology described by Smith et al. [29]. Post quality control, filtering and trimming, an Amplicon Sequence Variant (ASV) table was constructed, which was followed by the removal of chimeric sequences. Taxonomy was assigned to sequence variants using the SILVA database (v. 138.1) and phyla names were updated according to Oren and Garrity [30]. A bootstrapping threshold of 80 was applied to taxonomic classification by incorporating minBoot = 80 as part of the assignTaxonomy function [29]. Sample metadata, sequence taxonomy, and ASVs were combined into a phyloseq object using *Phyloseq* (v. 1.42.0) [31].

A rarefaction curve was plotted for colostral microbial samples. Based on the plateauing of the generated rarefaction curve (data available on OSF), sequencing was deemed to be conducted to a sufficient depth, and no rarefaction was performed. Following this, bacterial and archaeal ASVs, classified beyond the phylum level, were obtained. Datasets were recombined for the identification and removal of contaminants using a frequency- and prevalence-based approach with a threshold of 0.5 through *decontam* (v. 1.22.0) [32]. After removal of contaminants, data were separated by bacteria and archaea and analysed independently. Subsequently, alpha (α; Shannon) diversity was calculated for each sample. For comparisons of beta (β; composition) diversity and differential abundance analysis, the relative abundance (RA) of ASVs were calculated, and those that were not present in >0.05% were removed before further analysis. Beta diversity was depicted graphically using non-metric multidimensional scaling (NMDS) using Bray-Curtis plots. Due to low diversity and abundance, analysis of archaea was unable to proceed beyond taxonomic classification and α-diversity analysis.

### 2.5 Statistical analysis

#### 2.5.1 Colostrum quality.

Colostrum Ig concentrations (IgA, IgG and IgM), BRIX % and Shannon Index measures were analysed using SAS software (Version 9.4, SAS Institute Inc., Cary, NC, USA). Data were checked for normality and homogeneity of variance using the UNIVARIATE procedure. Using ANOVA (PROC MIXED procedure), the model included fixed effects of breed, parity, and their interactions. Differences between treatments were assessed using F-tests with Type III sums of squares, and treatment means were compared using the PDIFF command with Tukey’s test for multiple comparisons. Mean values were considered statistically significant at P ≤ 0.05. Non-significant terms (P > 0.05) were excluded from the final model.

#### 2.5.2 Sequencing data.

For analysis, animals were classified as primi- or multiparous based on lactation number. Prior to assessing the effect of breed, parity, and their interactions on overall prokaryotic community structure, the homogeneity of group dispersions was assessed between groups. Following this, PERMANOVA tests based on Bray-Curtis dissimilarities, 9,999 permutations and a significance level of P < 0.05 were implemented to determine if breed or parity affected prokaryotic community structure. Both assessment of homogeneity of group dispersions and PERMANOVA were conducted using *Vegan* (v. 2.6.4) [33]. Core bacteria identification and other analyses were performed using *Microbiome* [34], post-filtering for a RA of >0.05%. The core bacteria were defined as those taxa agglomerated at the genus level shared across colostrum samples [35]. All analysis was performed at the phylum and genus level due to poor species level classification. Statistical analysis of ASVs was performed using *MaAsLin2* (v. 1.14.1) [36] with parity and breed as fixed effects and the Benjamini-Hochberg procedure to correct for false discovery rate.

#### 2.5.3 Correlations.

Correlation analysis was performed to determine any potential associations between core bacteria, colostral ASVs, and colostrum quality measures. Additionally, correlation analysis was performed on the ASVs identified as core bacteria to explore potential relationships between the bacteria observed within this community. A Spearman’s rank-order correlation for non-parametric data was run using the *rcorr* function in *Hmisc* (v. 5.2.3) in *RStudio* (v. 4.3.0). Correlations (effect size and strength of the correlation, denoted as r) were described using the following: 0.00–0.19, “very weak”; 0.20–0.39, “weak”; 0.40–0.59, “moderate”; 0.60–0.79, “strong; and 0.80-1.00, “very strong” [37]. Only correlations with a P ≤ 0.05 were considered statistically significant.

## 3. Results

### 3.1 Colostrum quality

The coefficient of determination (R^2^) values for sRID plate analysis ranged from 0.97–1.00 (Data available on OSF). Overall, when incorporating genotype and parity effects in the statistical model, mean colostral IgA, IgG, and IgM concentrations did not differ (Table 2).

**Table 2:** Mean colostrum quality measures.

	All	SE^1^	Breed (B)			Parity (P)			P Value		
			Holstein	Jersey	Pooled SE^1^	Primi^2^	Multi^3^	Pooled SE^1^	B^4^	P^5^	P*B^6^
BRIX (%)	26.6	0.68	27.2	25.5	0.76	25.84	26.99	0.75	0.23	0.54	0.68
^7^IgA (g/L)	9.58	0.97	8.57	11.28	1.39	7.72	10.67	1.27	0.17	0.07	0.55
^8^IgG (g/L)	150.59	5.61	152.09	148.04	8.14	141.47	155.95	7.95	0.73	0.24	0.19
^9^IgM (g/L)	9.96	0.69	10.03	9.84	1.06	9.68	10.12	1.05	0.90	0.77	0.06

^1^SE: Standard Error; ^2^Primi: Primiparous; ^3^Multi: Multiparous; ^4^B: Breed; ^5^P: Parity; ^6^B*P: Breed × Parity interaction; ^7^IgA: Immunoglobulin A; ^8^IgG: Total Immunoglobulin G; ^9^IgM: Immunoglobulin M.

### 3.2 Sequencing performance and overall microbial composition

**Fig 2:**
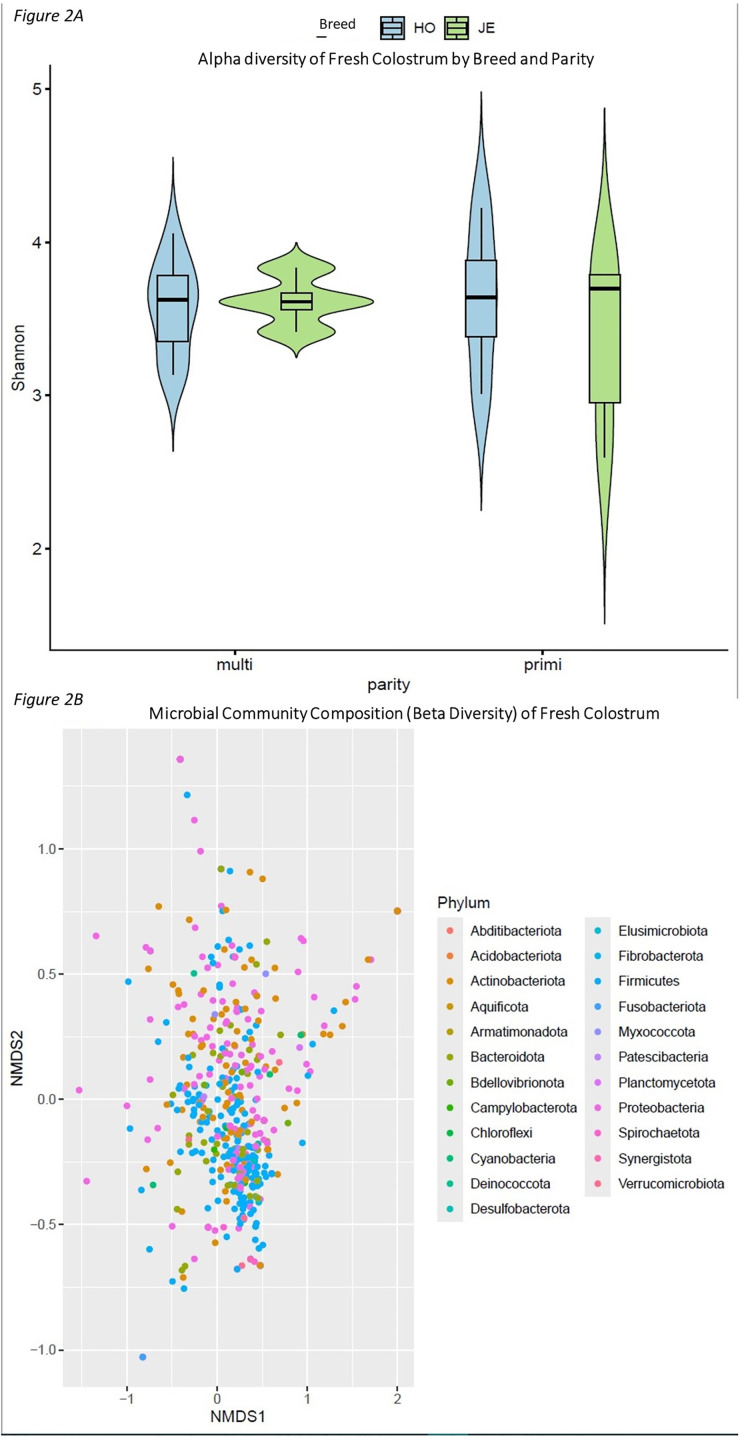
A: α-diversity (Shannon) of fresh colostrum by breed and parity. HO: Holstein-Friesian; JE: Jersey. B: Non-metric multidimensional scaling plot using Bray-Curtis dissimilarity to examine microbial composition in fresh colostrum.

After quality filtering, merging, removal of chimeric sequences and decontamination, an average of 81,782 (SE 7,201) reads per colostrum sample were generated, with a range of 28,285–135,201 reads. A total of 6,674 unique ASVs were identified after decontamination. When agglomerated by genus, this resulted in 681 different ASV groups, of which seven belonged to Archaea and the remaining 674 to Bacteria. Correlations between positive controls run for each kit were strongly correlated to one another with r ranging from 0.83–0.95 (P ≤ 0.01). Both breed and parity were found to be homogenous within group during assessment of homogeneity of group dispersions (parity: P = 0.45; breed: P = 0.43). Based on ANOVA results, no effects of breed (P = 0.4) or parity (P = 0.7) nor any interactions between the two, were observed on α-diversity (Shannon Index), and mean Shannon index score was 3.33 (SE 0.14) (Fig 2A). Based on PERMANOVA, breed (P = 0.14) and parity (P = 0.11), had no effect on the colostral prokaryotic community composition. Prokaryotic community composition of fresh colostrum was observed to be homogenous in nature (Fig 2B).

#### 3.2.1 Archaea.

Seven archaeal genera were identified, belonging primarily to the phylum Euryarchaeota (RA = 99.46%). The two most proportionally abundant genera were *Methanobrevibacter* (RA = 85.99%) and *Methanosphaera* (RA = 13.47%). Two additional genera, *Candidatus Methanogranum* (RA = 0.27%) and *Methanocorpusculum* (RA = 0.26%) were present in relative abundances greater than 0.05%. The remaining genera, *Sulophobococcus* (RA = 0.008%), *RumEn M2* (RA = 0.002%) and *Candidatus Nitrocosmicus* (RA < 0.001%) were lowly abundant and were not included in analysis post-filtering for a relative abundance of >0.05%.

#### 3.2.2 Bacteria.

**Fig 3:**
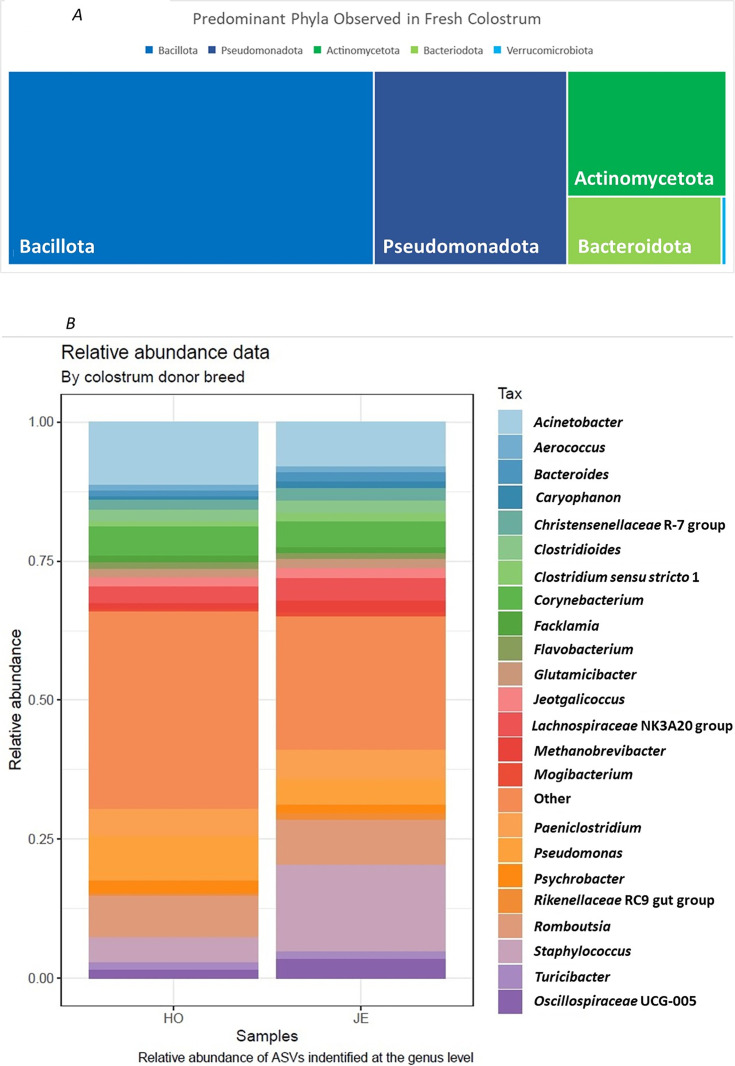
A: Top Five Phyla contributing to the bacterial component of the fresh colostral prokaryotic community. B Relative Abundance of predominant genera identified in fresh colostrum.

After filtering for a RA of >0.05% a total of 66 genera were observed to contribute to the bacterial component of the fresh colostrum microbiota (Fig 3B; additional data available on OSF). Five phyla were observed to be the predominant contributors: *Bacillota* (formerly Firmicutes; RA = 46.44%), *Pseudomonadota* (formerly Proteobacteria; RA = 19.15%), *Actinomycetota* (formerly Actinobacteria; RA = 14.37%), *Bacteroidota* (RA = 7.50%), and *Verrucomicrobiota* (RA = 0.26%) (Fig 3A).

Nineteen ASV groups, including nine of the 10 most proportionally abundant (RA = 46.25%; Fig 3B), were identified as core bacteria (Table 3). *Bacillota* (Firmicutes) accounted for the largest proportion of bacteria observed. Twenty-eight ASVs belonged to this phylum including *Staphylococcus* (RA = 4.21%), and 13 ASVs were observed to contribute to core bacteria. These core bacteria included *Romboutsia* (RA = 9.08%), *Christensenellaceae* R-7 group (RA = 2.07%), *Lachnospiraceae* NK3A20 group (RA = 3.52%) and *Clostridium sensu stricto* 1 (RA = 1.20%). *Pseudomonadota* (Proteobacteria) was the second most abundant phylum, with 10 ASVs observed as contributors to colostral bacteria. Four of these ASVs were identified as core bacteria: *Pseudomonas* (RA = 4.65%), *Acinetobacter* (RA = 9.70%), *Psychrobacter* (RA = 2.00%), and *Sphingomonas* (RA = 1.03%). Fifteen ASVs were identified as belonging to the phylum *Actinomycetota* (Actinobacteria), however, only one was identified as core: *Corynebacterium* (RA = 6.00%). Other genera identified in this phylum included *Dietzia* (RA = 0.32%), *Brachybacterium* (RA = 1.20%) and *Brevibacterium* (RA = 1.79%). *Bacteriodota* had 12 ASVs, with only *Bacteroides* (RA = 1.58%) identified as core bacteria. Additionally, *Flavobacterium* (RA = 1.22%), *Rikenellaceae* RC9 gut group (RA = 0.90%), *Alistipes* (RA = 0.74%), *Prevotellaceae UCG*-003 (RA = 0.38%) and *Prevotellaceae UCG-*004 (RA = 0.39%) were observed to contribute to the bacterial component of the fresh colostrum microbiota. Only one ASV belonging to the phylum *Verrucomicrobiota* was observed, *Akkermansia* (RA = 0.26%), however it was not identified as part of the core bacteria.

**Table 3:** Core bacterial genera of fresh colostrum, their relative abundance (RA) and metabolisms.

ASV^1^	Phylum	Family	Genus	RA^2^	Metabolism
ASV 1	*Pseudomonadota*	*Pseudomonadaceae*	*Pseudomonas*	4.65	Aerobe^3^
ASV 2	*Pseudomonadota*	*Moraxellaceae*	*Acinetobacter*	9.70	Aerobe
ASV 3	*Bacillota*	*Peptostreptococcaceae*	*Romboutsia*	9.08	Anaerobe^4^
ASV 5	*Bacillota*	*Peptostreptococcaceae*	*Paeniclostridium*	5.78	Anaerobe
ASV 10	*Bacillota*	*Peptostreptococcaceae*	*Clostridioides*	2.47	Anaerobe
ASV 11	*Bacillota*	*Staphylococcaceae*	*Jeotgalicoccus*	1.92	Facultative^5^
ASV 12	*Bacillota*	*Lachnospiraceae*	*NK3A20 group*	3.84	Anaerobe
ASV 13	*Actinomycetota*	*Corynebacteriaceae*	*Corynebacterium*	6.00	Facultative
ASV 15	*Pseudomonadota*	*Moraxellaceae*	*Psychrobacter*	2.00	Aerobe
ASV 16	*Bacillota*	*Aerococcaceae*	*Facklamia*	1.39	Facultative
ASV 17	*Bacillota*	*Aerococcaceae*	*Aerococcus*	1.33	Facultative
ASV 18	*Bacillota*	*Erysipelotrichaceae*	*Turicibacter*	1.54	Anaerobe
ASV 20	*Bacillota*	*Oscillospiraceae*	*UCG-005*	2.52	Anaerobe
ASV 24	*Bacillota*	*Clostridiaceae*	*Clostridium sensu stricto 1*	1.45	Anaerobe
ASV 25	*Pseudomonadota*	*Sphingomonadaceae*	*Sphingomonas*	1.03	Aerobe
ASV 29	*Bacillota*	*Planococcaceae*	*Caryophanon*	1.10	Facultative
ASV 32	*Bacteroidota*	*Bacteroidaceae*	*Bacteroides*	1.58	Anaerobe
ASV 53	*Bacillota*	*Christensenellaceae*	*R-7 group*	2.21	Anaerobe
ASV 81	*Bacillota*	*Monoglobaceae*	*Monoglobus*	0.60	Anaerobe

^*1*^*ASV: Amplicon Sequence Variant;* ^*2*^*RA: Relative Abundance, expressed as a percentage (%);* ^*3*^*Aerobe: denotes bacteria which are obligate aerobes, requiring oxygen to grow;* ^*4*^*Anaerobe: denotes bacteria which are obligate anaerobes and cannot survive in oxygenated environments;* ^*5*^*Facultative: denotes bacteria that are capable of function in an environment with or without oxygen.*

### 3.3 Correlations

No correlations with a P-value less than 0.05 were observed between IgA or IgM, other variables, or ASVs observed to be core. A moderate correlation was found between colostral IgG concentrations and BRIX % (r = 0.56, P = 0.003). Furthermore, one strong correlation and four moderate correlations were observed between donor lactation number and bacterial ASVs including *Clostridioides* (r = 0.45, P = 0.02). BRIX % was moderately correlated to five ASVs including *Lachnospiraceae* NK3A20 group (r = 0.45, P = 0.02). One moderate and two weak correlations were observed between colostral Ig concentrations and non-core ASVs (Data available on OSF).

**Fig 4:**
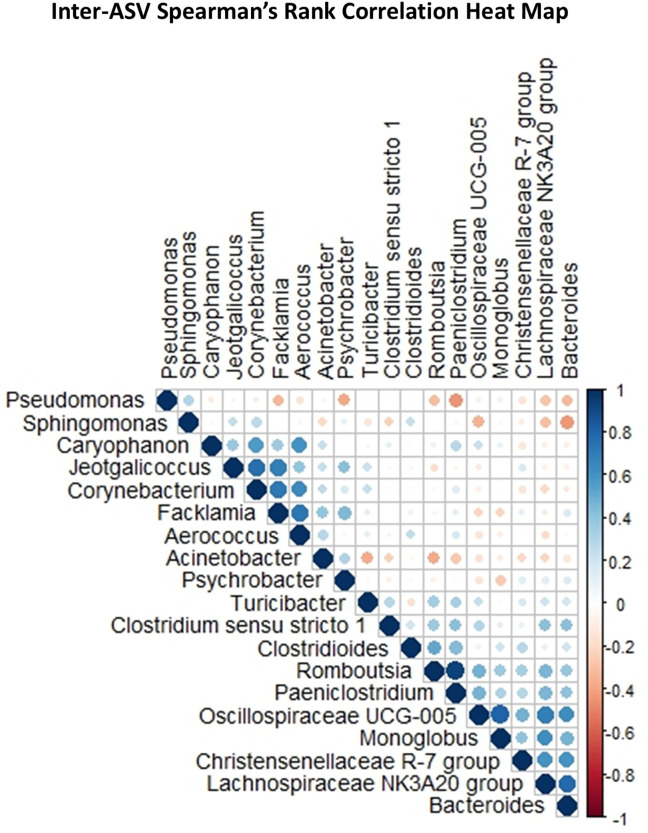
Heat-Map ordered by hierarchical clustering, demonstrating potential relationships between bacterial genera identified in fresh colostrum.

Several correlations were identified among the ASVs identified as core bacteria in fresh colostrum (Fig 4). A total of nine very strong correlations were observed, including correlations between *Bacteroides* and *Lachnospiraceae* NK3A20 group (r = 0.80, P < 0.0001), *Prevotellaceae UCG-003* and *Oscillospiraceae UCG-*005 (r = 0.82, P < 0.0001) and *Paeniclostridium* and *Romboutsia* (r = 0.92. P < 0.0001). Eighty-six strong correlations were observed, including correlations between *Lachnospiraceae* NK3A20 and *Oscillospiraceae UCG-*005 (r = 0.70, P = 0.0001), *Monoglobus* (r = 0.68, P = 0.0001) and *Christensenellaceae* R-7 gut group (r = 0.60, P = 0.001). *Christensenellaceae* R-7 gut group was also strongly correlated to *Oscillospiraceae* NK4A214 group (r = 0.66, P = 0.0002) and *Oscillospiraceae UCG-005* was strongly correlated to *Bacteroides* (r = 0.70, P < 0.0001). Two hundred and fifteen moderate correlations were observed between bacterial community members, including positive correlations between *Romboutsia* and *Clostridioides* (r = 0.54, P = 0.004), *Lachnospiraceae* NK3A20 group (r = 0.44, P = 0.02) and *Oscillospiraceae* UCG-005 (r = 0.47, P = 0.01) (full list of correlations available on OSF).

## 4. Discussion

The present study observed a diverse yet homogenous bacterial community within colostrum with no effect of breed or parity. The presence of archaea and common bovine gut commensals is indicative of a complex microbial community. The variety of correlations observed within the bacterial community provides insight into relationships between the bacteria present. Future work should focus on understanding the function of these microbes, their interactions with each other and the host, and the impact this may have on the calf.

### 4.1 Colostrum quality

Colostrum IgG concentrations (150.59 g/L) observed in the present study, indicate that the colostrum sampled was of excellent quality. Colostrum quality is measured through direct or indirect quantification of IgG concentrations, where quality defined as ‘good’ has IgG present in quantities of >50 g/L (direct) or having a BRIX reading of ≥ 22% (indirect) [2,38]. Colostral Ig concentrations are influenced by farm management practices, calving season, breed, and parity [2,39]. In the present study, no effect of breed or parity on Ig concentrations was observed. However, comparative research indicates variations in colostrum immunoglobulin concentrations of different dairy breeds [40]. Muller and Ellinger [41] investigated the colostral Ig concentration of five different cattle breeds, including Ayrshire, Brown Swiss, Guernsey, Holstein-Friesian, and Jersey cows, and observed that Jersey cows consistently had the greatest IgG, IgA, and IgM concentrations. The authors acknowledge that the lack of variation in Ig concentrations may be associated with sample size, which was determined by calculating sample size for sufficient power for microbiome analysis and is in line with the good research practice of minimizing the use of animals.

Colostral IgA and IgM concentrations in bovine colostrum have previously been reported at mean values of 1.66–4.40 g/L and 4.20–4.90 g/L, respectively [40,42]. Both IgA and IgM are synthesized locally in the mammary gland [43] and are present in proportionally lesser quantities than IgG [2]. These immunoglobulins play a vital role, functioning as additional immune support for the neonatal calf [10]. IgA accumulates at the epithelial surface [2], helps prevent pathogens from attaching to the epithelium [44] and is an essential part of the ‘kill zone’ portion of the mucosal barrier [10]. In the present study mean IgA and IgM concentrations are 9.58 g/L and 9.96 g/L, respectively. Taking into consideration IgA and IgM concentrations when assessing colostrum quality, the concentrations reported in the present study supports the assertion that the colostrum sampled was of excellent quality.

### 4.2 The colostral prokaryotic community

Breed has previously been shown to influence the composition of various bovine microbiota compositions, including the rumen and faecal microbiota. However, lack of breed effect on the colostrum microbiota is similar to previous findings reported by Scully et al. [24]. While investigating the faecal microbiota in dairy heifer calves during the pre-weaning period, no effect of breed was observed either. In another study examining cow-calf contact and rumen microbiota development during the pre-weaning period, there was no observed difference between Holstein and Montbeliarde calves, likely because these calves were all home-bred and reared on the same farm [45]. No effect of breed on the diversity and composition of the colostral microbiota supports the assertion that an effect of farm origin may be presenting as an effect of breed in microbiota studies, as has previously been suggested by Scully et al. [24].

#### 4.2.1 Archaea.

The majority of archaea observed belonged to the phylum *Euryarchaeota*, known as methanogens, that are frequently reported as normal members of the ruminal microbiota in cattle [46,47]. Within this phylum, *Methanobrevibacter* and *Methanosphaera* were the major contributors to the archaeal component of the fresh colostral microbiota. Archaea are prokaryotic microbes found in diverse environments, including as commensal microbes in the mammalian gastrointestinal tract [46,48]. They are strictly anaerobic [47] and play key roles in nutrient cycling and methanogenesis [46]. Hoque et al. [49] previously reported the presence of archaea in milk, including the two genera observed in the present study. Additionally, Huuki et al. [12], Vasquez et al. [16] and Zhu et al. [13] also detected *Methanobrevibacte*r in colostrum.

These two genera have been previously identified as the primary archaeal genera found in calf faeces [12] and the small intestine of the neonatal calf [50]. In a meta-analysis of bovine gastrointestinal microbiome studies, Holman and Gzyl [51], found that *Methanobrevibacter* and *Methanosphaera* were present in all rumens, rumen epithelium, and faecal samples analysed. The detection of archaea, particularly these two genera, in colostrum supports the hypothesis of a bovine colostral microbiome and the existence of an entero-mammary pathway in ruminants. This finding also supports the theory that colostral prokaryotes may be involved in the seeding of the archaeal community of the calf rumen and hindgut microbiomes, as suggested by Huuki et al. [12].

Both *Methanobrevibacter* and *Methanosphaera* are primary contributors to the archaeal component of the rumen microbiota [47]. Previous studies have associated the presence of these genera in the rumen with low methane emissions in ruminant livestock [52–55]. As the rumen community stabilizes around day 21 post-birth [20], any attempts to modulate this community should occur before this time. Future research should focus on the role of colostral archaea in seeding and colonization of the rumen and hindgut, as well as potential applications to early life modulation, enteric methane emissions, and methane mitigation.

#### 4.2.2 Bacteria.

The existence of an entero-mammary pathway, where gut microbes are mobilized from the gastrointestinal tract and transported to the mammary through the blood by dendritic cells during colostro- and lactogenesis [56,57] has already been established in humans and mice [15,56]. Examining the biological mechanisms associated with an entero-mammary pathway was beyond the scope of the current study, however, key microbes reported in the present study, does support the transfer of microbes from one body site to another. Four of the five phyla observed, *Bacillota* (Firmicutes), *Pseudomonadota* (Proteobacteria), *Bacteriodota* and *Actinomycetota* (Actinobacteria), have also been reported in other colostrum microbiota [6,13,16,18,19,58,59] and milk microbiome studies [1,4,49,60]. They also contribute to the udder [14], vaginal [14,60], and rumen [21,58] microbiomes of adult cattle and the faecal microbiota of calves and adults [12,24,50,61,62]. *Verrucomicrobiota* was also identified as a contributor to the bacterial component of the colostral microbiota in the present study. This phylum has previously been observed in colostrum [18], faeces [12,24] and in rumen digesta samples [20], further supporting the possibility of an entero-mammary pathway and the transfer of microbes between body sites.

At the genus level, the current study identified 19 bacterial genera as members of the core bacteria. In microbial ecology, core refers to microbiome characteristics that are shared across a set of samples, a host, or an environment [35]. Core microbiome/microbiota can be quantified in different ways depending on the data set and the specificities being investigated [35]. For the purposes of this study “core” refers to those bacterial ASVs, which were identified and agglomerated at the genus level, present in a RA of >0.05% and prevalent in 90% of the samples. Chen et al. [19] also investigated the core bacteria in colostrum sourced from two different Chinese dairy farms and, in agreement with the current study, identified 21 different genera as core members of the colostral prokaryotic community. Both this study and Chen et al. [19] found *Acinetobacter, Bacteroides, Christensenellaceae* R-7 gut group, *Paeniclostridium, Pseudomonas, Psychrobacter, Romboutsia, and Sphingomonas* contribute to the core bacteria observed in colostrum. Additionally, Chen et al. [20] found that *Eubacterium, Bacillus, Brevundimonas, Chryseobacterium, Paracoccus, Staphylococcus* and *Rikenellaceae* RC9 gut group to be core bacteria members. In the present study, these genera were present in colostrum at relative abundances greater than 0.05% but were not classified as core bacteria. These and many of the other bacteria identified in the present study (i.e., *Corynebacterium, Streptococcus, Enterococcus*, *Lactobacillus*) have also been identified in other colostral microbiota studies [6,12,13,18,59]. *Acinetobacter, Bacteroides, Corynebacterium, Pseudomonas, Staphylococcus* and other genera previously mentioned have also been reported in milk microbiome studies [15,49,60,63]. To the authors’ knowledge, the present study is the first to report the observation *Lachnospiraceae* NK3A20 and *Oscillospiraceae UCG-*005 in colostrum, both of which were observed to be core bacterial community members.

Many of the bacterial genera that were observed in the present study are commonly observed in the bovine gastrointestinal microbiota, including the rumen, hindgut and faeces of calves and mature cattle [12,50,51]. Several of these genera are obligate anaerobes, including *Akkermansia, Prevotellaceae UCG-*003*, Prevotellaceae UCG-*004*, Clostridium sensu stricto* 1 and *Mogibacterium* (a full list of genera observed in fresh colostrum and various bovine microbiome studies available on OSF). It is possible that some of these microbes originated from the teat/udder [5,15] or other maternal sources [13,14,58]. However, the presence of archaea (*Methanobrevibacter*), obligate anaerobes (*Akkermansia* etc.) and common gut commensals (*Christensenellaceae* R-7 gut group*, Rikenellaceae* RC9 gut group*, Oscillospiraceae UCG-005* etc.) suggests the presence of a complex bovine colostral microbiome, as has been previously suggested by Lima et al. [6] and Taponen et al. [60]. All these prokaryotes are strict anaerobes, and their presence warrant further research into the mechanisms behind their presence in colostrum.

### 4.3 Implications for calf health

#### 4.3.1 Immunological associations.

Castillo-Lopez et al. [64] reported that when fed colostrum, calf immunoglobulin concentration was positively correlated to the abundance of *Lactobacillaceae* and *Lachnospiraceae* observed in calf faeces. This supports findings by Van Hese et al. [18] who reported a positive correlation between colostrum quality, calf passive transfer of immunity and the bacterial family *Lachnospiraceae*. In the present study, there was a moderate positive correlation between BRIX refractometer scores (indirect measure of colostrum quality) and *Lachnospiraceae*. *Lachnospiraceae* was observed to have a variety of positive correlations with other core bacteria, ranging from moderate to very strong, including *Romboutsia,* *Oscillospiraceae* and *Christensenellaceae,* all bovine gut commensals that have been previously associated with good gut health in calves [24]. This, in combination with similar findings from the aforementioned studies, suggests that *Lachnospiraceae* may have potential to be used as a microbial biomarker indicative of good quality colostrum with a prokaryotic profile that may benefit calf health. The correlations observed between this family and the other ASVs, mainly known bovine gut commensals, warrants further investigation into the potential to use it as an indicator of a favourable colostrum microbiota profile for calf hindgut microbiome modulation to enhance enteric health during the neonatal period.

#### 4.3.2 Intestinal development.

Both Yeoman et al. [14] and Zhu et al. [13] concluded that the colostral microbiota is a maternal source of microbes, providing initial gut colonizers from the dam to the calf. Initial gut colonizers are essential in transitioning the hindgut from an aerobic to an anaerobic environment, which allows for proper microbial colonization and development. In the present study, the colostral prokaryotic community was found to consist of 23 aerobic and 23 facultatively anaerobic bacteria, all of which could actively contribute to transitioning the hindgut environment. Some of the bacteria present in colostrum may also produce short chain fatty acids. Several of the genera observed in the present study are known to produce propionate, acetate and butyrate. Butyrate is known to play a key role in the growth and development of epithelial cells in the hindgut [51]. The epithelial cells of the hindgut are not only essential to nutrient absorption, but they are also critical in the formation and maintenance of the mucosal immune system of the host [10]. Fan et al. [65] found that butyrate producing microbes in the bovine hindgut were directly associated with various genes responsible for the regulation of host immunity. Acetate and propionate are both involved in host immune maintenance and are potentially involved in the gut-lung axis and immune response during respiratory disease [66]. The present study, along with their potential functions and the findings of previous studies, highlight the need for further research on the systemic implication of a colostral microbiome and the potential role in neonatal calf development.

### 4.4 Limitations of the study

The author’s acknowledge the small sample size in this study as a limitation. While 27 samples were sufficient to characterise the archaeal and bacterial components of the colostrum microbiota within a single pasture-based dairy herd, the findings may not be generalisable to other herds or production systems. The objective of the study was to jointly characterise the archaeal and bacterial components of the colostrum microbiota from a single pasture-based dairy herd. The herd comprised both Holstein-Friesians and Jerseys with varying parities, and although breed and parity were considered during analysis, the study was not powered to detect differences between these groups. The findings reported in this study highlight the need for more in-depth investigation into the colostrum microbiota, and the potential of this community in the development of calf probiotics and microbiota modulation. Additionally, there is a need to research the impact of colostrum management practices on this community and calf health.

## 5. Conclusions

This study demonstrates that fresh colostrum collected directly after calving harbours a diverse and homogeneous microbial community, largely composed of genera known as bovine gut commensals. While the existence of an entero-mammary pathway remains inconclusive, the findings suggest possible maternal microbial transfer and highlight *Lachnospiraceae* as a potential biomarker for colostrum quality. These results underscore the relevance of colostral microbiota as a foundation for neonatal gut development and point to promising applications in probiotic design. Future research should determine the viability and functional role of these microorganisms, as well as the influence of colostrum management practices on both microbial communities and calf health outcomes.
